# Discrepancies between explicit and implicit evaluation of aesthetic perception ability in individuals with autism: a potential way to improve social functioning

**DOI:** 10.1186/s40359-020-00437-x

**Published:** 2020-07-10

**Authors:** Monica Mazza, Maria Chiara Pino, Roberto Vagnetti, Sara Peretti, Marco Valenti, Antonella Marchetti, Cinzia Di Dio

**Affiliations:** 1 Department of Applied Clinical Sciences and Biotechnology, University of L’Aquila, Via Vetoio, Località Coppito, 67100 L’Aquila, Italy; 2 Reference Regional Centre for Autism, Abruzzo Region Health System, L’Aquila, Italy; 3 Department of Psychology, University Cattolica del Sacro Cuore, Milan, Italy

**Keywords:** Aesthetic perception, Empathy, Social cognition, Autism spectrum disorder, Eye-tracking

## Abstract

**Background:**

The capacity to evaluate beauty plays a crucial role in social behaviour and social relationships. It is known that some characteristics of beauty are important social cues that can induce stereotypes or promote different behavioural expectations. Another crucial capacity for success in social interactions is empathy, i.e. the ability to understand and share others’ mental and emotional states. Individuals with Autism Spectrum Disorder (ASD) have an impairment of empathic ability. We showed in a previous study that empathy and aesthetic perception abilities closely related. Indeed, beauty can affect different aspects of empathic behaviour, and empathy can mediate the aesthetic perception in typically developing (TD) individuals. Thus, this study evaluates the ability of aesthetic perception in ASD individuals compared to TD individuals, using the Golden Beauty behavioural task adapted for eye-tracking in order to acquire both explicit and implicit evidences. In both groups, the relationship between empathic and aesthetic perception abilities was also evaluated.

**Methods:**

Ten ASD individuals (age ± SD:20.7 ± 4.64) and ten TD individuals (age ± SD:20.17 ± 0.98) participated in the study. Participants underwent empathy tasks and then the Golden Beauty task. To assess differences in the participants’ performance, we carried out a repeated measures general linear model.

**Results:**

At the explicit level, our behavioural results show an impairment in aesthetic perception ability in ASD individuals. This inability could have relevance for their ability to experience pleasure during social interactions. However, at the implicit level (eye-tracking results), ASD individuals conserved a good ability to feel aesthetic pleasure during the Golden Beauty task, thus indicating a discrepancy between the explicit and implicit evaluation of the beauty task. Finally, beauty perception appears to be linked to empathy when neither of these capacities is compromised, as demonstrated in the TD group. In contrast, this link is missed in ASD individuals.

**Conclusion:**

Overall, our results clearly show that individuals with autism are not completely blind to aesthetic pleasure: in fact, they retain an implicit ability to experience beauty. These findings could pave the way for the development of new protocols to rehabilitate ASD social functioning, exploiting their conserved implicit aesthetic perception.

## Background

In non-human primates, aesthetic perception plays a crucial role in mate selection and reproductive capacities [1, 2]. In the human species, the ability to perceive beauty has an additional relevance in influencing social behaviour [3]. Across different cultures, there exist features of beauty that determine an ‘objective beauty’; at the same time, beauty can induce a ‘subjective pleasure’ in each person [4, 5]. In fact, human aesthetic judgement is a complex mix of genetic, cultural, objective and subjective factors [1]. It has indeed been shown that more attractive women have more offspring over a lifetime compared to less attractive women. In addition, some features of faces, like symmetry, are generally associated with fertility and even higher moral values [3, 6]. In bargaining, attractive people receive higher offers [7] and tend to be considered as more reliable, even by children [8], supporting a strongly rooted proclivity to aesthetics. Judgement of other people’s attractiveness probably occurs subconsciously and influences us in ways we do not consciously realise [3]. Taken together, these findings suggest that some characteristics of beauty are important social cues that can induce stereotypes or promote different behavioural expectations [9]. Ultimately, they may also affect the ability to experience pleasure, which plays an important role in social interactions [10, 11]. A fundamental capacity for successful social interactions is social cognition (SC), a complex cognitive construct that allows one to encode and decode the social world [12, 13].

Autism Spectrum Disorder (ASD) is a neurodevelopmental disorder characterised by a) deficit in social communication and social interaction, and b) restricted, repetitive patterns of behaviours, interests or activities [14]; its prevalence in the general population is around 1% [15, 16]. It is known that people with ASD show impairment of SC abilities [12]. Specifically, people with autism have difficulty with the ability to experience empathy, which is a main component of SC. Empathy should no longer be considered as a unitary concept, but is a multidimensional process that includes at least two dimensions [17–20]: a cognitive component (also known as theory of mind), consisting of the ability to understand and explain the mental states of others—in other words, what others are thinking or feeling [17]; and an emotional component, being the ability to respond emotionally to other people’s feelings while understanding that they are distinct from one’s own [21, 22]. A third component of empathy is the motor dimension, which allows one to align with others’ visible behaviour and to understand the associated emotional state on the basis of personal experience—namely, to empathise [23, 24]. Empathic ability is crucial in human interactions because it allows one to automatically understand and share the actions and internal states of others. Researchers have suggested that empathic abilities are related to aesthetic perception. Along these lines, the aesthetic experience of artworks has been proposed to consist of the activation of embodied simulation of actions, as well as of corporeal and emotional sensations. Embodied simulation consists of a mirroring mechanism which constitutes a basic functional mechanism in social cognition [25]. Moreover, embodied theories of the aesthetic experience argue that the content of the artwork (which could be intended as actions, intentions, objects, emotions and sensations) drives a simulation based on our mirror mechanism [25]. The term ‘simulation’ refers here to an automatic and unconscious mechanism which aims to interpret intentions from the overt behaviour of others by generating a representational content; moreover, it serves to attribute to others actions, emotions or sensations [26]. For example, it has been demonstrated that the observation of classical and Renaissance sculptures activates a motor mirroring congruent with the movement represented in the sculpture [27]. Thus, the component of empathy (cognitive, emotional and motor) shows an overlap with embodied simulation theories. In this respect, it is worth noting that mirror systems have been understood as a substantial part of the basic functional mechanism in SC in terms of embodied simulation [26, 27], therefore supporting an important role of its components, among which is empathy in aesthetic experiences [25, 27], In fact, the aesthetic experience felt by the viewer is the reflection of the artist’s intention to convey a specific emotional state, and the quality of the relationship between the observer and the artwork is established by the emotions and sensations reflected [25]. The link between aesthetic experience and emotions is also supported by neuroimaging studies [24, 28–31], which have shown that the perception of beauty is mediated by the activity of cerebral areas also involved in empathic ability. Di Dio et al. [28] showed that the observation of classical sculptures, compared to the same sculptures with modified proportion between body parts, induced joint activation of lateral and medial cortical areas (lateral occipital gyrus, precuneus and prefrontal areas), responding to the physical properties of the stimuli, and the anterior insula. In particular, the aesthetic experience induced by canonical art could emerge from the processing of sensorimotor input in conjunction with the emotional feeling of pleasure that is mediated by activation of the insula [5]. In this respect, it is worth noting that the anterior portion of the insular cortex has most often been associated with tasks involving empathic engagement with others’ feelings or sensations [32–35] and is activated when there is an emotional mirror resonance [23]. In addition, it has been shown that tasks requiring an aesthetic judgement often activate the same reward network in the brain that responds to the sensory pleasures associated with love, food and drugs via dopaminergic pathways [36, 37]. It is also known that the reward system is active in general empathic behaviour, and especially prosocial behaviour such as cooperation [37, 38]. Taken together, these data suggest a relationship between aesthetic and empathic abilities, in that beauty can affect different aspects of empathic behaviour, just as empathic abilities can partly mediate aesthetic perception [13]. Of particular relevance, evidences show that viewing artwork could elicit mirroring mechanisms and areas involved in empathic abilities [28], and empathic areas are elicited during aesthetic judgements [28]; moreover a positive association has been found between empathy and aesthetic judgement [13], although it must be pointed out that the nature of the relationships still needs to be fully understood. Moreover, it has been proposed that embodied simulation could play an important role during the aesthetic experience, in which simulation could also allow understanding of intentions.

As ASD are characterised by impaired SC [12], of which empathy is an important component, and a dysfunctional insula anterior connectivity has been found [39], ASD’s aesthetic perception could suggest how the impairment of this dimension could lead to a different aesthetic experience. Moreover, we hypothesise that ASD’s aesthetic perception would result in less interest and pleasure gained by viewing the art, despite objectively recognising it as beautiful Moreover, we administer empathy tasks to verify ASD’s impairment and to understand the relationship between this dimension and its components in the aesthetic experience for the ASD and the TD sample respectively. Furthermore, the eye-tracking technique was used to gather additional information about sensory-driven coding of the stimuli by assessing eye movement behaviour.

## Methods

### Participants

The study included 20 participants: ten subjects with ASD, selected by the Reference Regional Centre for Autism of L’Aquila, Abruzzo Region (ASD group, mean age ± SD:20.7 ± 4.64), and ten TD subjects (TD group, mean age ± SD:20.17 ± 0.98). The TD subjects were recruited from the University of L’Aquila.

The ASD group presented uneven distribution by gender (nine males and one female); thus we matched the TD group by gender (nine males and one female). No differences between groups (ASD and TD) emerged for chronologic age (t_1_._19_ = 0.27; *p* = 0.78). On the basis of chronological age, individuals with ASD were tested with the Wechsler Adult Intelligence Scale (WAIS-IV) [39]. The ASD diagnosis was provided by experienced clinicians according to the new criteria of the DSM-5 [14] and was confirmed using the Autism Diagnostic Observation Schedule, Second Edition (ADOS-2) [40]. Exclusion criteria were a) impaired cognition (assessed by WAIS-IV); b) presence of comorbidity; and c) presence of drug treatment. As the experiment was assessed in a clinical setting through exclusion criteria, we only managed to achieve ten ASD participants; thus we recruited ten TD participants in order to have equal sample sizes. Socio-demographic and clinical information on the two groups of participants are summarised in Table 1.

**Table 1:** Between-groups differences for demographic data, clinical information and empathy measures

	ASD (*N* = 10) Mean (SD)	TD (*N* = 10) Mean (SD)	t (df = 1, 19)	*P*
Chronological age (in years)	20.70(4.64)	20.17 (0.98)	0.27	0.78
**Clinical information**				
ADOS-social communication and social interaction	10.00 (4.25)	–	–	–
ADOS- Repetitive and Stereotyped Behaviours	1.20 (1.13)	–	–	–
ADOS Total scores	11.90 (3.81)	–	–	–
QIV	98.00 (23.27)	103.40 (19.72)	−0.549	0.590
QIP	95.00 (13.50)	95.60 (12.34)	−0.104	0.919
QIT	96.40 (15.23)	97.40 (13.03)	−0.158	0.876
**Empathy measures**				
*Advanced Theory of Mind task*	7.60 (3.37)	12.67 (0.51)	−3.60	**0.003**
*Eyes-task*	17.90 (3.84)	28.50 (3.01)	−5.75	**0.0001**
*Basic Empathy Scale*				
Affective Empathy sub-scale	33.30 (6.76)	42.00 (3.68)	−2.87	**0.012**
Cognitive Empathy sub-scale	28.40 (5.40)	40.67 (3.44)	−4.45	**0.0001**
*Empathy Quotient*				
Cognitive empathy sub-scale	8.30 (1.88)	16.33 (3.20)	−6.37	**0.0001**
Social skill sub-scale	4.00 (0.81)	10.83 (1.32)	−12.85	**0.0001**
Emotional empathy sub-scale	9.40 (3.59)	16.66 (3.26)	−4.04	**0.001**

Significant comparisons are highlighted in bold

### Procedure

The ASD and TD groups performed the same test sessions. The participants were evaluated in two sessions. During the first session, the participants completed the empathy-related tests (Eyes task, Basic Empathy Scale, Empathy Quotient, Advanced Theory of Mind task) using paper and pencil measures. This session lasted approximately 50 min. During the second session, the participants performed the aesthetic perception task called Golden Beauty (see below for more details) using Tobii T120 eye tracker and E-Prime® Extensions for Tobii Pro™ for simultaneous behavioural data acquisition. The eye-tracking session lasted 20 min. All the subjects were mother tongue Italian and had normal or corrected to normal vision. Moreover, all participants were untrained in the arts. The participants were tested individually in a quiet room according to the principles established by the Declaration of Helsinki. The investigation was approved by the Ethical Committee of the NHS Local Health Unit (Azienda Sanitaria Locale 1) that approved the experimental protocol prior to the recruitment of participants, according to the principles established by the Declaration of Helsinki. Informed consent in written form was obtained from all the participants before the study.

### Empathy measures

#### Eyes task [41]

The Eyes Task is a revised version of the Reading the Mind in the Eyes test. This test was considered by Baron-Cohen and collaborators [41] as a ‘pure’ theory of mind test. The participants were given 36 photographs depicting the ocular area in an equal number of different actors and actresses. At each corner of every photo, four emotional descriptors (e.g. dispirited, bored, playful or comforting) were printed, only one of which (the target word) correctly identified the depicted person’s mental state, the others being included as foils. The overall score was obtained by totalling the number of items (emotions) that the participant correctly identified. Therefore, the maximum total score is 36.

#### Basic empathy scale (BES) [42, 43]

The BES is composed of two subscales: the affective empathy subscale (AES) and the cognitive empathy subscale (CES). The AES is composed of 11 items that measure the ability to share another person’s emotions. An example of items in the AES is: “My friend’s emotions don’t affect me much”. The participants were asked to give their ratings on a five-point Likert-type scale ranging from 1 (strongly disagree) to 5 (strongly agree). The CES comprises nine items and measures understanding of another person’s emotion [42]. Examples of items in the CES are: “I can understand my friend’s happiness when she/he does well at something” and “When someone is feeling down, I can usually understand how they feel”.

#### Empathy quotient (EQ) [44]

The EQ is a self-report measure evaluating different aspects of empathy through cognitive, social skill and emotional subscales. The cognitive dimension of empathy is evaluated by two subscales of the EQ: cognitive empathy (CEQ) and social skills (SSQ), which measure, respectively, the capacity to take the perspective of the other person, and some regulatory mechanisms that keep track of the origins of one’s own and other’s feelings. The emotional dimension is evaluated by the emotional subscale (EEQ). An example of items is “I find it hard to understand how to behave in a social situation”. Each answer can vary from 0 (strongly agree) to 4 (strongly disagree).

#### Advanced theory of mind task (A-ToM) [45]

The A-ToM is an Italian adaptation of a cognitive task that Blair and Cipolotti [46] used, first proposed by Happè [43]. The Italian task consisted of a short version of 13 vignettes, each accompanied by two questions: the comprehension question (“Was it true, what X said?”) and the justification question “(Why did X say that?”). The 12 story types include Lie, White Lie, Joke, Pretence, Misunderstanding, Double Bluff, Contrary Emotions, Figure of Speech, Appearance/Reality, Forgetting, Irony and Persuasion. Each subject obtained a score ranging from 0 to 1 for each question. The maximum score was 13. Happè [45] used the term ‘advanced’ to refer to a story that contains the comprehension question, where the key questions in the task concern a character’s mental state (the experimental condition).

### Golden beauty task

To evaluate aesthetic perception, we used a modified version of the Golden Beauty (GB) task [28]. The GB task consists of the evaluation of images of sculptures selected from masterpieces of classical and Renaissance art that are commonly accepted as normative Western representations of beauty [28]. This task evaluates the sense of beauty through an objective parameter (proportion) intrinsic to specific works of art, and requires an objective and a subjective aesthetic judgement, as well as a proportion judgement for proportioned and modified stimuli. The main feature of this task is the use of two sets of stimuli that are identical in every respect but one: proportion. Specifically, a parameter that is considered to represent ideal beauty specifically in the classical representation of the human body [47, 48] was modified to create an aesthetically degraded version of the same stimuli in a controlled fashion. The GB task contained 44 images of sculptures, including 22 images with modified proportions between body parts. In particular, half of the canonical stimuli were modified with a short-leg long-trunk relationship, and the other half with the opposite modification. All stimuli (canonical and modified sculptures) were presented in three experimental conditions: 1) objective aesthetic judgement (OAJ) for proportioned stimuli (OAJP) and modified stimuli (OAJM); 2) subjective aesthetic judgement (SAJ) for proportioned stimuli (SAJP) and modified stimuli (SAJM); and 3) proportion judgement (PJ) for proportioned stimuli (PJP) and modified stimuli (PJM). In the first condition (OAJ), participants were asked to observe the sculptures and to express an explicit objective judgement (“objective” aesthetic value) for each image by answering the question: “Is the image you see beautiful?” The participants responded on a dichotomous scale: Yes = it is beautiful, or No = it is not beautiful. In the second condition (SAJ), participants were asked to observe the sculptures and to express an explicit subjective judgement (“subjective” aesthetic value) for each image by answering the question: “Do you like the image that you see?” The participants responded on a dichotomous scale: Yes = I like it, or No = I don’t like it. In the third condition (PJ), participants were asked to observe the sculptures and to express an explicit proportion judgement for each image by answering the question: “Is the image that you see proportional?” The participants responded on a dichotomous scale: Yes = It is proportional or No = It is not proportional. (An example of a Golden Beauty task in reported in Additional file 1).

### Eye-tracker stimuli and settings

The GB experiment was performed using E-Prime® Extensions for Tobii Pro™ and Tobii T120 Eye Tracker equipment consisting of a GL-2760-LED backlight monitor with resolution of 1920 × 1080 pixels, on which the stimuli were presented and from which gaze behaviour was recorded simultaneously. The eye-tracking system is non-invasive, with little indication that eye movements are being tracked, and artificially constrained head movements are not required. The system tracks both eyes to a rated accuracy of 0.5 degrees with a sampling rate of 60 Hz. The Tobii equipment was connected to a Lenovo laptop computer (Windows 7 Professional) that was used to run the tasks. The two blocks of the GB task—i.e. objective and subjective aesthetic judgements (OAJ and SAJ, respectively)—were administered in a random order, while the block of proportion judgement (PJ) was always presented at the end of the task for all participants so as not to influence the subjects’ aesthetic evaluation with prior exposure to the proportion assessment. All participants were seated facing the eye-tracker monitor at a distance of roughly 70 cm, with the experimenter sitting next to the subject to control the computer screen without interfering with observation of the images. Detailed instructions were provided just before the experimental session; the instructions were again presented on the screen at the beginning of each test block. The participants looked at a total of 132 stimuli (sculptures) divided into 44 stimuli (22 proportionate and 22 modified) for each block (OAJ, SAJ, PJ). The presentation of each stimulus lasted 4 s. The subjects were asked to look at the stimuli (sculptures) as they were presented on the screen, and then a task-related question about the stimulus appeared, which was associated with one of the three blocks (OAJ: Is the image that you see beautiful? SAJ: Do you like the image that you see? PJ: Is the image that you see proportional?). The duration of the question slide was 5 s. A calibration test consisting of five registration points was performed before the GB task. The calibration was repeated if one of the five points was not valid. During the calibration phase, the participants were asked to visually follow a small red ball presented on the screen. Calibration procedures, stimulus creation, data acquisition and visualisation were performed using Tobii Studio™ analysis software. The behavioural data were acquired using E-Prime® Extensions for Tobii Pro™.

### Eye-tracker data acquisition and model analysis

Data were collected using Tobii Studio™. For each of the stimuli of the GB task, areas of interest (AOI) were drawn to investigate fixations to specific regions of the human body. A total of four AOI (face, arms, trunk and legs) were created to have fixations of all parts of body.

Two gaze parameters were analysed: time to first fixation—the time from when the stimulus was shown until the start of the first fixation within an AOI; and total fixation duration*—*the sum of all fixations’ duration recorded within an AOI. A fixation event was defined as such by the Tobii fixation filter (I-IV filter) when the point of gaze remained within 0.5 degrees of a visual angle for at least 100 milliseconds. Data for each AOI were normalised with respect to the total area of the image. Total fixation duration indicates the time for which an AOI is viewed, thus giving a measure of how attention is given to the stimulus, while time to first fixation indicates the time from the AOI appearing on the screen to its viewing. Thus a lower time to first fixation time of subject A compared to time of first fixation of subject B indicates that subject A views that specific AOI before subject B, and a reduced time to first fixation between stimuli in the same subject indicates which AOI has been viewed before others.

### Data analysis and results

All of the continuous variables were normally distributed with skewness between − 1 and 1. The homogeneity of variance was checked for all parametric tests, and eventual corrections are reported. For the factorial analyses, the Greenhouse-Geisser correction was used for violations of Mauchy’s test of sphericity (*p* = .05). All multiple comparisons were Bonferroni-adjusted (*p* = .05).

### Empathy measures

We used independent t-test analyses to compare the ASD and TD groups on empathy scores. The results showed that the ASD group was impaired in all empathy measures (Eyes task, BES, EQ, and A-ToM task) compared to the TD group.

Specifically, regarding the Eyes task, the ASD group obtained a lower score (t_1,19_ = − 5.75; *p* = 0.0001) compared to the TD group; similarly, individuals whit autism showed difficulties in both the AES (t_1,19_ = − 2.87; *p* = 0.01) and CES (t_1,19_ = − 4.95; *p* = 0.0001) subscales of BES compared to the TD group. Additionally, the ASD group received lower scores compared to the TD group in the EEQ (t_1,19_ = − 4.04; *p* = 0.001), CEQ (t_1,19_ = − 6.37; *p* = 0.0001), and SSQ (t_1,19_ = − 12.85; *p* = 0.0001) subscales of EQ. Finally, ASD individuals showed impaired performance in the A-ToM task (t_1,19_ = − 3.60; *p* = 0.003) compared to the TD group. The results of these analyses are reported in Table 1.

### Behavioural data

#### Aesthetic judgements

To assess differences in the participants’ attributions of aesthetic preference in the two aesthetic tasks (objective and subjective) for the canonical and modified stimuli, we carried out a repeated measures general linear model (GLM), with two levels of task type (OAJ and SAJ) and two levels of stimulus type (canonical and modified) as the within-subject factors, and group (ASD and TD) as the between-subject factor. The results showed a main effect of stimulus type (canonical>modified; F_1,18_ = 21.07, *p* = .0001, partial-η^2^ = .53, δ = .99), as well as significant interactions between stimulus type and group (F_1,18_ = 10.09, *p* = .005, partial-η^2^ = .36, δ = .85). Additionally, the results showed a significant difference in performance between the two groups, which was independent of the other factors (TD > ASD; F_1,18_ = 30.71, *p* = .0001, partial-η2 = .63, δ = .99). A post-hoc analysis (Bonferroni-corrected) showed that the interaction between stimulus type and group stemmed from the lack of significance between canonical and modified stimuli in the ASD group (M_diff_ = .80, SE = .80, *p* = .33), whereas this difference was significant for the TD group (canonical>modified; M_diff_ = 4.40, SE = .80, *p* = .0001; see Table 2).

**Table 2:** Mean differences between canonical and modified stimuli in aesthetic and proportion judgments task for both groups (ASD and TD), separately

Golden Beauty task	Group	(I) Canonical stimuli type Mean (SE)	(J) Modified stimuli type Mean (SE)	Mean_diff_ (I-J) (SE)	*P*
Aesthetic Judgment task	ASD group	10.300 (.715)	9.500 (.431)	.800 (.801)	.331
	TD group	15.500 (.715)	11.100 (.431)	4.400 (.801)	**.0001**
Proportion judgment task	ASD group	14.500 (.962)	13.000 (1.301)	1.500 (.922)	.121
	TD group	19.300 (.962)	8.900 (1.301)	10.400 (.922)	**.0001**

Significant comparisons are highlighted in bold

#### Proportion judgement

To assess differences in the participants’ evaluation of proportion in the two stimulus types (canonical and modified) between the ASD and TD groups, we carried out a repeated measures GLM. The results showed a main effect of stimulus type (canonical>modified; F_1,18_ = 83.35, *p* = .0001, partial-η^2^ = .82, δ = .1), as well as significant interactions between stimulus type and group (F_1,18 =_ 46.62, *p* = .0001, partial-η^2^ = .72, δ = 1). Assessing the interaction effect revealed that there was no difference in proportion evaluation between canonical and modified stimuli in the ASD group (M_diff_ = 1.50, SE = .92, *p* = .12), whereas this difference was significant for the TD group (canonical>modified; M_diff_ = 10.40, SE = .92, *p* = .0001; see Table 2).

#### Correlation between empathy measures and conditions of golden beauty task

Pearson’s correlations were computed to assess the relationships between the conditions of the GB task (OAJP-OAJM, SAJP-SAJM, PJP-PJM) and the empathy measures (Eyes task, BES, EQ and A-ToM task).

#### ASD group

No significant correlations were found between any of the empathy measures and the conditions in the GB task for the ASD group.

#### TD group

For the TD group, significant correlations were found between the OAJP, SAJP and PJM conditions of GB and the Eyes task, the CEQ subscale of EQ, and both subscales (AES, CES) of BES. Specifically, a significant positive correlation was found between the OAJP condition of the GB task and the Eyes task (*r* = .711; *p* = .022). The SAJP of the GB task positively correlated with both the AES (*r* = .251; *p* = .032) and CES (*r* = .707; *p* = .034) subscales of BES. Finally, we found significant negative correlations between the PJM of the GB task and CES (*r* = −.849; *p* = .033) subscale of BES, the CEQ (*r* = −.889; *p* = .020) subscale of EQ, and the Eyes task (*r* = −.888; *p* = .018).

#### Summary of behavioural results

The behavioural findings associated with the empathic measures used in the study (Eyes task, BES, EQ and A-ToM) showed that individuals with autism are impaired in all measures of empathy (and their subscales). Moreover, in the TD group, but not in the ASD group, we found that GB objective, subjective and proportion tasks correlated with three empathy measures—i.e. BES (AES and CES subscales), EQ (CEQ subscale), and the Eyes task. Specifically, in the TD group, cognitive empathic ability, as measured by the Eyes task, correlated with a good ability to give an objective judgement of beauty for the proportional sculptures (OAJP). In addition, the capacity to evaluate their subjective pleasure for proportional sculptures (SAJP) correlated with affective and cognitive empathic ability, measured through the AES and CES subscales of BES. Finally, the ability to evaluate as less proportional the sculptures with modified proportions between body parts correlated with cognitive empathic ability (measured by the Eyes task, CES subscale of BES and CEQ subscale of EQ). This is in line with the idea that proportion evaluation can be regarded as the perceptual-cognitive component of the task [28]. On the whole, these results suggest that perception of beauty appears to be linked with empathy when these capacities are not compromised. Additionally, our results indicated that people with ASD also show impairment in aesthetic perception ability. In fact, the ASD group had lower ability to judge as objectively beautiful and subjectively pleasing (aesthetic judgements) both canonical and modified sculptures, compared to TD people. At the same time, there were no differences in the ASD group in the evaluation of proportion for the canonical and modified sculptures (proportion judgement). In contrast, the TD group evaluated the canonical sculptures as more proportional compared to the modified stimuli. In addition, the TD group evaluated the sculptures with canonical proportions as more pleasing and beautiful (subjective and objective judgements, respectively) and more proportional (proportion judgement) compared to the sculptures with modified proportions.

### Eye-tracking data

To assess differences in the participants’ observation pattern during GB tasks (objective aesthetic task, subjective aesthetic task and proportion evaluation task), for each eye-tracking parameter (time to first fixation and total fixation duration), repeated measures GLM analyses were carried out with four levels of AOI (body parts: face, arms, trunk and legs) and two levels of stimulus type (canonical and modified sculptures) as the within-subject factors, and group (ASD and TD) as the between-subject factor.

### Objective aesthetic task

**Fig. 1:**
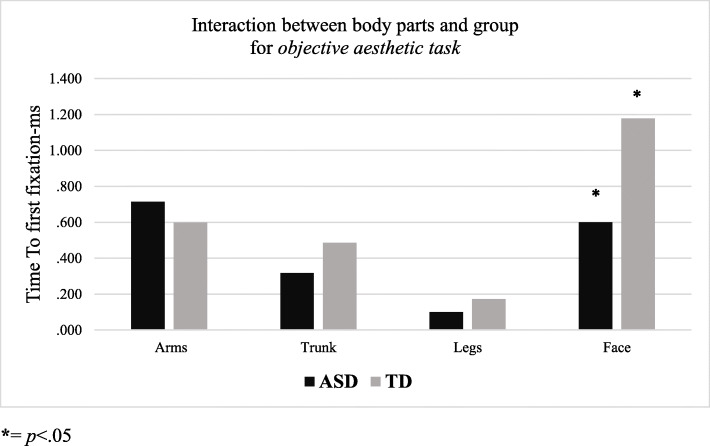
Significant interaction between body parts (arms, trunk, legs, face) and group (ASD and TD) for *objective aesthetic task* in relation to the time to first fixation (in milliseconds). * = *p* < .05

With respect to time to first fixation, the results showed a main effect of body parts (legs > other body parts; F_1,18_ = 17.88, *p* = .0001, partial-η^2^ = .49, δ = .1), as well as significant interactions between body parts and group (F_1,18_ = 3.63, *p* = .01, partial-η^2^ = .16, δ = 76). Assessing the interaction effect, we found that the interaction between body parts and group was significant for the face area (*p* = .05; see Table 3 and Fig. 1) in the ASD group.

**Table 3:** Descriptive statistics and mean differences between groups (ASD and TD) of eye-tracking parameters (time to first fixation and total fixation duration in milliseconds) in objective aesthetic task for all body parts (arms, trunk, legs, face)

ET-parameters	Task	Body Parts	(I) ASD group Mean (SE)	(J) TD group Mean (SE)	Mean_diff (J-)_ (SE)	*P*
Time to First Fixation	Objective Aesthetic task	Arms	.714 (.151)	.599 (.151)	.115 (.214)	.598
		Trunk	.316 (.90)	.485 (.090)	−.169 (.127)	.202
		Legs	.101 (.059)	.172 (.054)	−.071 (.076)	.359
		Face	.600 (.188)	1.178 (.188)	−.578 (.266)	**.044**
Total Fixation Duration	Objective Aesthetic task	Arms	.629 (.103)	.612 (.103)	.018 (.145)	.905
		Trunk	.326 (.088)	.478 (.088)	−.153 (.125)	.238
		Legs	.099 (.052)	.158 (.052)	−.058 (.073)	.437
		Face	.590 (.189)	1.173 (.189)	−.583 (.268)	**.043**

Significant comparisons are highlighted in bold

**Fig. 2:**
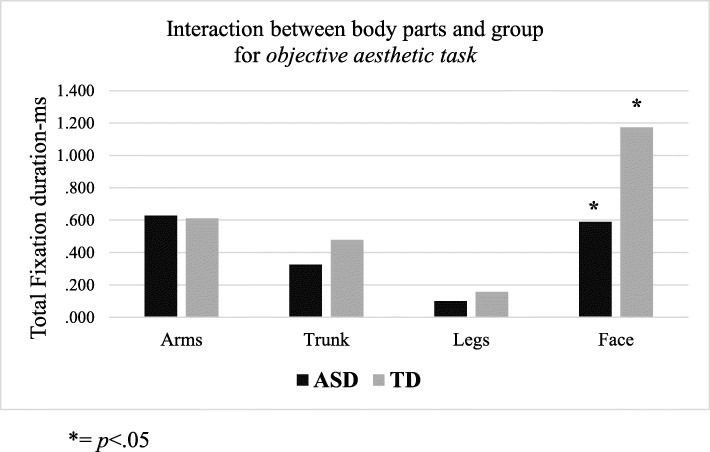
Significant interaction between body parts (arms, trunk, legs, face) and group (ASD and TD) for *objective aesthetic task* in relation to the total fixation duration (in milliseconds). * = *p* < .05

With respect to total fixation duration, the results showed a main effect of body parts (face>other body parts; F_1,18_ = 22.54, *p* = .0001, partial-η^2^ = .55, δ = .1), as well as a significant interaction between body parts and group (F_1,18_ = 3.96, *p* = .01, partial-η^2^ = .18, δ = 80). The interaction stemmed from significantly longer total fixation duration on the face area in the TD group, but not in the ASD group, compared to the other body parts (*p* = .05; see Table 3 and Fig. 2).

#### Subjective aesthetic task

The results for time to first fixation (TFF) and total fixation duration (TFD) showed a main effect of body parts (legs>other body parts; TFF: F_1,18_ = 26.89, *p* = .0001, partial-η^2^ = .59, δ = 1; arms>other body parts TFD: F_1,18_ = 27.79, *p* = .0001, partial-η^2^ = .60, δ = 1), indicating that both groups looked first at the legs and longer at the arms compared to other body parts (*p* = .05; see Table 4). No differences were found between groups with respect to time to first fixation and total fixation duration.

**Table 4:** Mean differences in eye-tracking parameters (time to first fixation and total fixation duration in milliseconds) between body parts (arms, trunk, legs, face) during the subjective aesthetic task, in both groups (ASD and TD)

ET-parameters	Task	(I) Body parts	(J) Body parts	M_diff_ (I-J) (SE)	*P*
Time to First Fixation	Subjective Aesthetic task	Arms	Trunk	.325 (.055)	**.0001**
			Legs	.574(.075)	**.0001**
			Face	.122 (.067)	.525
		Trunk	Legs	.250 (.040)	**.0001**
			Face	−.203 (.069)	.054
		Legs	Face	−.453 (.093)	**.001**
Total Fixation Duration	Subjective Aesthetic task	Arms	Trunk	.269 (.049)	**.0001**
			Legs	.525 (.0.62)	**.0001**
			Face	.066 (.075)	1.00
		Trunk	Legs	.256 (.042)	**.0001**
			Face	−.203 (.058)	**.015**
		Legs	Face	−.459 (.085)	**.0001**

Significant comparisons are highlighted in bold

#### Proportion evaluation task

The results for time to first fixation showed a main effect of body parts (legs>other body parts; F_1,18_ = 21.33, *p* = .0001, partial-η^2^ = .54, δ = 1). Indeed, both groups look first at the legs compared to other body parts (*p* = 05; see Table 5). No differences were found between groups with respect to time to first fixation.

**Table 5:** Mean differences in time to first fixation (in milliseconds) between body parts (arms, trunk, legs, face) during the subjective aesthetic task, in both groups (ASD and TD)

ET-parameters	Task	(I) Body parts	(J) Body parts	M_diff_ (I-J) (SE)	*P*
Time to First Fixation	Proportion evaluation task	Arms	Trunk	.343 (.072)	**.001**
			Legs	.567 (.069)	**.0001**
			Face	−.077 (.126)	1.00
		Trunk	Legs	.224 (.036)	**.0001**
			Face	−.420 (.094)	**.002**
		Legs	Face	−.644 (.122)	**.0001**

Significant comparisons are highlighted in bold

**Fig. 3:**
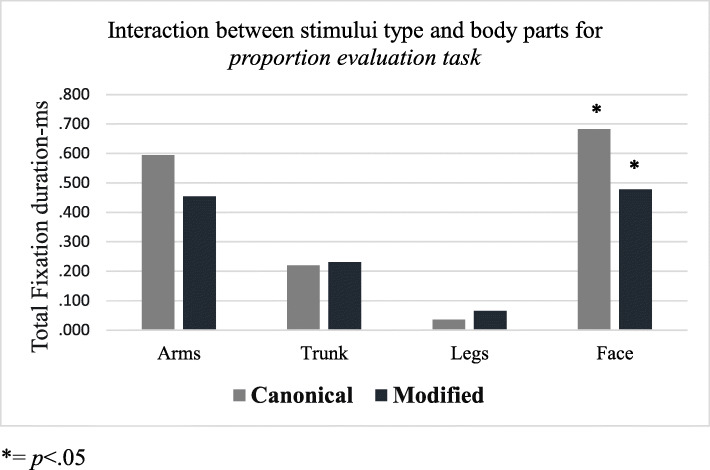
Significant interaction between body parts (arms, trunk, legs, face) and stimuli type (canonical and modified) for *proportion evaluation task* in relation to the total fixation duration (in milliseconds). * = *p* < .05

Regarding total fixation duration, the results showed a main effect of stimulus type (canonical>modified; F_1,18_ = 7.94, *p* = .01, partial-η^2^ = .30, δ = .76), a main effect of body parts (face>other body parts; F_1,18_ = 25.27, *p* = .0001, partial-η^2^ = .58, δ = 1), as well as a significant interaction between stimulus type and body parts (F_1,18_ = 3.66, *p* = .01, partial-η^2^ = .16, δ = .77). Additionally, the results showed a significant difference in performance between the two groups (TD mean = .434; SE = .057) > ASD mean = .257; SE = .057) F_1,18_ = 4.77, *p* = .04, partial-η^2^ = .21, δ = .54), suggesting that the TD group generally fixated longer than the ASD group. A post-hoc analysis showed that the interaction between stimulus type and body parts lack of significant differences between canonical and modified stimuli in terms of total fixation duration on the arms (M_diff_ = .14, SE = .07, *p* = .07), trunk (M_diff_ = .01, SE = .04, *p* = .81) and legs (M_diff_ = .03, SE = .01, *p* = .11), whereas this difference was significant for the face area (M_diff_ = .20, SE = .07, *p* = .01) in both groups. That is, participants generally fixated longer on the face area of the canonical than the modified stimuli (*p* = .05; see Table 6 and Fig. 3).

**Table 6:** Descriptive statistics and mean differences in total fixation duration (milliseconds) between stimuli type (canonical and modified) for all body parts (arms, trunk, legs, face), during the proportion evaluation task in both groups (ASD and TD)

ET-parameters	Task	Body Parts	(I) Canonical stimuli type Mean (SE)	(J) Modified stimuli type group Mean (SE)	Mean_diff_ (I-J) (SE)	*P*
Total Fixation Duration	Proportion evaluation task	Arms	.594 (.078)	.454 (.052)	.140 (.075)	.078
		Trunk	.220 (.053)	.230 (.037)	−.010 (.042)	.811
		Legs	.036 (.015)	.066 (.018)	−.030 (.018)	.116
		Face	.683 (.117)	.477 (.088)	.206 (.078)	**.016**

Significant comparisons are highlighted in bold

#### Summary of the eye-tracking results

The eye-tracking results showed no differences between groups in the observation pattern for the subjective aesthetic judgement task. On the other hand, in the objective aesthetic judgement task, the TD group, but not the ASD group, fixated significantly longer on the face compared to the other body parts of both canonical and modified sculptures. Additionally, we found that the ASD group achieved a lower score (total fixation duration) for the face of all sculptures compared to the TD group.

According to research in this field [27, 49, 50], during their visual exploratory behaviour, observers concentrate their gaze on specific areas of an image. The parts of an image with longer fixation durations were interpreted as indicating the observer’s interest in informative elements of the image [27, 51]. Additionally, it is known that eye movements are the expression of the relationship between what is looked at and its importance to the observer’s interest [52]. Longer fixation time for the TD individuals indicates that faces were of more interest to them compared to the ADS group. Faces are complex visual stimuli that have a special role in social perception and SC because they convey important information for effective interpersonal interactions and non-verbal communication [53]. It is well known that face perception is atypical in people with ASD [53–55]. This is also supported by the longer total fixation duration found for the TD group. As shown in Massaro et al. [27] and Savazzi et al. [56], when a human subject is depicted in an image, regardless of other contextual cues possibly present in the representation, the viewer’s attention is automatically drawn towards exploration of the face area. The pre-rational visual search within the image towards the face, determines longer time to first fixation to the face compared to representations in which this element is lacking and for which any point of the image can be a potential area of attraction. Faces represent a rather complex feature for ASD individuals to process and, taken together, our results suggest that the ASD group produced lower scores in all gaze parameters associated with faces because of their atypical processing of faces.

However, in the proportion evaluation task, both groups (ASD and TD) produced longer total fixation durations on the faces of canonical sculptures compared to both the modified stimuli and the other body parts. This was plausibly due to the fact that, when proportion was preserved, the face area represented the most interesting area of scrutiny. Conversely, when proportion was altered, as in the modified sculptures, attention was oriented towards the parts of the body that underwent modification, namely legs and trunk. Comparable exploration patterns found for the objective aesthetic and proportion evaluation tasks support the idea that the objective appreciation of artworks it is closely connected to perceptual recognition of the physical properties of the stimuli (i.e. proportion) [28, 30].

In sum, our results support the idea that, when people with autism give a judgement of objective beauty or subjective pleasure, they do not rely on the face to evaluate beauty, as appears to be the case for TD individuals. However, when ASD individuals evaluate the proportions (more perceptual-cognitive condition), they explore the whole body including the face if the sculpture has canonical proportions between body parts, although, at the level of behavioural performance, the ASD group was less skilled at assessing proportions compared to the TD group (see behavioural results above).

Finally, a significant difference between groups was found in the proportion evaluation task, with the TD group producing greater scores for both gaze parameters (time to first fixation and total fixation duration) compared to the ASD group. This result suggests that ASD individuals’ attention to the stimuli was lower during the proportion evaluation task compared to the TD group, possibly affecting ASD disproportion identification, as shown by the behavioural results (PJP task).

## Discussion

This study shows for the first time that individuals with ASD are impaired in their aesthetic perception ability. We used the Golden Beauty task, which aims at evaluating an individual’s sense of beauty through the use of an objective parameter intrinsic to classical works of art—i.e. proportion—and that requires objective and subjective aesthetic judgements of proportioned and proportion-modified stimuli, as well as a proportion evaluation of the same stimuli [28].

In recent years, interest in aesthetic perception ability within social behaviour has grown, as well as in understanding its fundamental role in improving or avoiding social interaction [3]. Besides the ability to appreciate aesthetics, two other abilities are crucial for successful social interaction: social cognition and empathy [13, 57]. In this respect, several investigations have consistently pointed out that aesthetic perception ability involves or shares part of the neural network underlying empathic abilities [23, 25, 28, 58]. It has been shown in neuroimaging studies that beauty perception, using the Golden Beauty task, produces joint activation of the cortical areas involved in the physical description of the stimulus, in a matching process between the external stimulus and one’s inner representation of it, and, crucially, activation of the anterior insular cortex [28, 30]. This latter structure has also been shown to be strongly involved in empathic abilities in TD individuals [32, 33, 58]. Additionally, beauty perception seems to be related to activation of the reward network in the brain [35], which is also active during different empathic behaviours (prosocial interactions and cooperation) [38]. According to De Ridder and Vanneste [58], beauty can be defined as a domain mediating the relationship between a positive response of the reward system and the experience of pleasure (hedonic). Altogether, these data indicate that, in typically developing individuals, empathic capacities are related to aesthetic perception, allowing one to anticipate, promote or obstruct the social interactions of others [3, 11]. In consideration of autistic individuals’ impairment in social interactions and empathic abilities [12, 19, 59], to date there is a significant lack of research aimed at assessing aesthetic perception abilities in autism. For this reason, in this study we evaluated aesthetic ability in an ASD group compared to a TD group, with the aim of confirming ASD impairment in these competencies, as well as assessing the relation between empathy and aesthetic perception.

Our behavioural results for different measures of empathy (Eyes task, BES, EQ, A-ToM) confirmed the findings of previous studies [12, 17, 58] and showed that people with ASD, compared to the TD group, have difficulties in both cognitive and affective empathy. As is known, these deficiencies affect ASD individuals’ ability to understand and share others’ emotions and mental states, resulting in the inability to engage in adequate social behaviour with other people [12, 20]. With respect to the GB task, our behavioural result showed that individuals with autism have a lower capacity to evaluate objectively-defined beauty (at least in the Western culture) compared to the TD group. We have previously suggested that ‘beauty’ can be defined as an important social factor creating positive or negative expectations about relationships with others and, moreover, promotion or avoidance of interactions with other people [1, 13, 60]. Therefore, impairment in aesthetic perception ability and in empathic abilities observed in individuals with autism could negatively strengthen their inability to be socially adequate. Moreover, our results regarding the subjective aesthetic judgement condition (subjective pleasure) showed that the ASD group always had lower subjective pleasure evaluations compared to the evaluation of objective beauty and proportion of sculptures with both canonical and modified proportion compared to the TD group. Beauty has a hedonic value, and, for this reason, it is strongly linked to the capacity to experience pleasure [58]. The opposite of hedonic—i.e. anhedonia—is a symptom of some psychiatric conditions (such as schizophrenia). Anhedonia consists of a decreased capacity to experience pleasure through those things that usually induce pleasure (such as attractive individuals or pleasant relationships), and this negatively affects the ability to experience interpersonal and social pleasure [11]. Based on that, our results may suggest that ASD individuals’ functional impairment in specific processing abilities, as outlined above, may affect their capacity to perceive aesthetics, which may ultimately compromise their ability to experience subjective pleasure with a consequent impact on social interactions.

Additionally, in this study, we gathered information about sensory-driven coding of the stimuli, exploring eye movement behaviour during the Golden Beauty task. The eye-tracking results showed that individuals with autism obtained lower scores (lower total fixation duration) for the face area of both modified and canonical sculptures in the objective aesthetic judgement task compared to the TD group. No differences between groups were found in terms of observation pattern for the subjective aesthetic judgement task. As already mentioned above, these results indicate that the parts of an image with shorter fixation duration can be interpreted as indicating the observer’s lack of interest in informative elements of the image [27, 51, 56]. In our specific case, data showed that people with autism also have difficulties evaluating objective beauty compared to TD individuals at an implicit level (eye-movement behaviour). The ASD group showed that faces were not a salient visual aspect of the artwork during the objective aesthetic judgement task, as opposed to the TD group that, on the other hand, paid great attention to the face area when judging the aesthetic of the stimulus. It is known that beauty perception is linked to the ability to appreciate or neglect faces based on their level of attractiveness, as suggested by studies showing that beauty perception shares the same neural network devoted to face processing [3, 58], involving areas such as the fusiform gyrus [31, 61–65]. Individuals with autism notably have difficulties processing faces. According to Pavlova et al. [53], for example, atypical face processing in individuals with autism could be due to their difficulties with visual integration—i.e. global perceptual ability [53]. Additionally, poor attention to faces in the ASD group may possibly depend on the fact that faces are stimuli carrying information about emotions, non-verbal communication and personality [53]. This latter point is also worth noting in consideration of the eye-tracking results associated with the proportion evaluation task in the present study. With respect to the proportion evaluation task, we found in both groups longer total fixation duration on the face of canonical sculptures compared to other body parts and modified stimuli. Crucially, no differences were found between groups in the exploration pattern of the face region, contrary to what was found during the aesthetic judgement tasks, in which ASD individuals did not consider the face to evaluate the beauty of the stimulus, while the TD group did. Proportion evaluation is the perceptual-cognitive component of the GB task, whereas both aesthetic judgement tasks involve an emotional component. The fact that ASD individuals did attend to the face area when judging proportion—namely, when there was no emotional involvement—suggests that avoiding looking at the face region is strongly related to ASD individuals’ impairment in processing emotions [12, 66]. ASD difficulty with emotions has relevance not only for beauty processing, as our data suggest, but also for empathic abilities, both fundamental components during social interaction. This idea is further supported by the correlations between cognitive and affective empathy measures and all conditions of the Golden Beauty task. Our results, in fact, showed significant correlations in the TD group but not in the ASD group, in agreement with previous research investigating empathic and aesthetic abilities, and showing that empathic and aesthetic perception abilities influence each other [58]. However, where these abilities are impaired, as in individuals with autism, this relationship is lost or, at least compromised.

Though promising, the current results are limited by the small sample size of the groups (ASD and TD). Regarding the sample size, we are aware that the statistical power in the various measures’ ranges between values of weak power (the minimum statistical power found was 51%, while the highest power found was 76%). This, of course, constitutes a serious problem of the study, but it is worth stressing that our results are highly suggestive, as we have found differences, and so the issue merits further study. However, it is also important to note that the power analysis of observed outcomes could be analytically misleading [67, 68]. Accordingly, further studies are needed with larger samples to strengthen the robustness of our findings. Additionally, future investigations could aim at studying aesthetic perception in individuals with ASD using other kinds of beauty tasks in order to outline more general differences in beauty processing, and to produce thereafter a complete aesthetic protocol that could improve (habilitate/ rehabilitate) beauty perception abilities in people with autism.

## Conclusion

Our findings suggest that individuals with autism are impaired in their ability to evaluate beauty, at least when beauty is associated with an objective parameter intrinsic to works of art (proportion in the case of classical representations). Their incapacity to process aesthetic features may have relevance in influencing the capacity to experience and recognise interpersonal and social pleasure, with a significant negative impact on their already compromised social interaction capacities. Concluding, this novel way of looking at general social abilities could represent an important tool for deepening knowledge of the typical clinical profile observed in ASD individuals. Moreover, this new concept could be useful in the design of individualised intervention goals for beauty and empathy abilities in order to improve the quality of life and social behaviour of ASD young adults.

## Supplementary information

**Additional file 1.** A: represents a sculpture with canonical proportions between body parts. B: represents a sculpture with modified proportions between body parts.

## Data Availability

The datasets generated and/or analysed during the current study are not publicly available as they contain sensitive material. The Ethical Committee of the NHS Local Health Unit (Azienda Sanitaria Locale 1) does not allow database sharing and we have to seek additional approval from the local ethics committee. However, they are available from the corresponding author on a reasonable request.

## Abbreviations

ASD: Autism Spectrum Disorder
TD: Typically developing
SC: Social cognition
GB: Golden Beauty
OAJ of GB: Objective aesthetic judgement
OAJP of GB: Objective aesthetic judgement for proportioned stimuli
OAJM of GB: Objective aesthetic judgement for modified stimuli
SAJ of GB: Subjective aesthetic judgement
SAJP of GB: Subjective aesthetic judgement for proportioned stimuli
SAJM of GB: Subjective aesthetic judgement for modified stimuli
PJ of GB: Proportion judgement
PJP of GB: Proportion judgement for proportioned stimuli
PJM of GB: Proportion judgement for modified stimuli
A-ToM: Advanced Theory of Mind task
EQ: Empathy quotient
CEQ of EQ: Cognitive empathy subscale
SSQ of EQ: Social skills subscale
EEQ of EQ: Emotional subscale
BES: Basic empathy scale
AES of BES: Affective empathy subscale
CES of BES: Cognitive empathy subscale
TFF: Time to first fixation
TFD: Total fixation duration
