# Supplementary material for: Discrepancies between explicit and implicit evaluation of aesthetic perception ability in individuals with autism: a potential way to improve social functioning
Source: BMC Psychol. 2020 Jul 10;8:74. doi: 10.1186/s40359-020-00437-x (PMC7350653; doi:10.1186/s40359-020-00437-x)
Supplement: Supplementary file 1 — Additional file 1. A: represents a sculpture with canonical proportions between body parts. B: represents a sculpture with modified proportions between body parts. [file 40359_2020_437_MOESM1_ESM.docx]

**Additional file 1.**

**A**: represents a sculpture with canonical proportions between body parts

**B**: represents a sculpture with modified proportions between body parts
